# Patterns and Correlates of Sedentary Behavior and Physical Activity in Individuals With Crohn’s Disease: A Cross-Sectional Study

**DOI:** 10.1093/crocol/otaf042

**Published:** 2025-06-28

**Authors:** Jason J Wilson, Barry Lynch, Nathan Graham, Conor M McClean, Mark A Tully

**Affiliations:** School of Sport and Exercise Science, Ulster University, Derry-Londonderry Campus, Londonderry, UK; School of Sport and Exercise Science, Ulster University, Derry-Londonderry Campus, Londonderry, UK; School of Sport and Exercise Science, Ulster University, Derry-Londonderry Campus, Londonderry, UK; School of Sport and Exercise Science, Ulster University, Belfast, Antrim, UK; School of Medicine, Ulster University, Derry-Londonderry Campus, Londonderry, UK

**Keywords:** Crohn’s disease, physical activity, sedentary behavior

## Abstract

**Background:**

Evidence suggests that being physically active could offer a range of benefits for people living with Crohn’s disease. However, the extent to which physical activity may provide benefits in terms of quality of life, mental health, and well-being requires further elucidation. This study aimed to highlight patterns and explore the correlates of sedentary behavior and physical activity in individuals living with Crohn’s disease.

**Methods:**

Adults living with Crohn’s disease from Ireland and the United Kingdom completed an online survey. Participants completed questions on: demographic characteristics; physical activity; sedentary behavior; Crohn’s disease severity; quality of life; anxiety and depressive symptoms; and mental well-being. Multiple linear regression analysis explored the correlates of sedentary behavior and physical activity.

**Results:**

One-hundred and eleven individuals (median age = 40.0 [31.0-48.0] years; 77% female) completed the survey. For sedentary behavior, median time was 9.14 (7.43-11.25) hours/day and the only significant correlate was age (β = −0.07, *t*(107) = −2.65, *P* = .01). For total physical activity, the quality of life physical health domain was the only significant correlate (β = 29.14, *t*(107) = 2.53, *P* = .01).

**Conclusions:**

Higher levels of sedentary behavior were associated with lower age, potentially due to the type of occupations of younger participants (ie, office-based jobs). Higher total physical activity levels were associated with higher quality of life physical health domain scores, which demonstrates the potential role physical activity might have in improving quality of life in individuals living with Crohn’s disease. Both sedentary behavior and physical activity might be beneficial lifestyle variables to target for health improvement in this population.

## Graphical Abstract

**Graphical Abstract:**
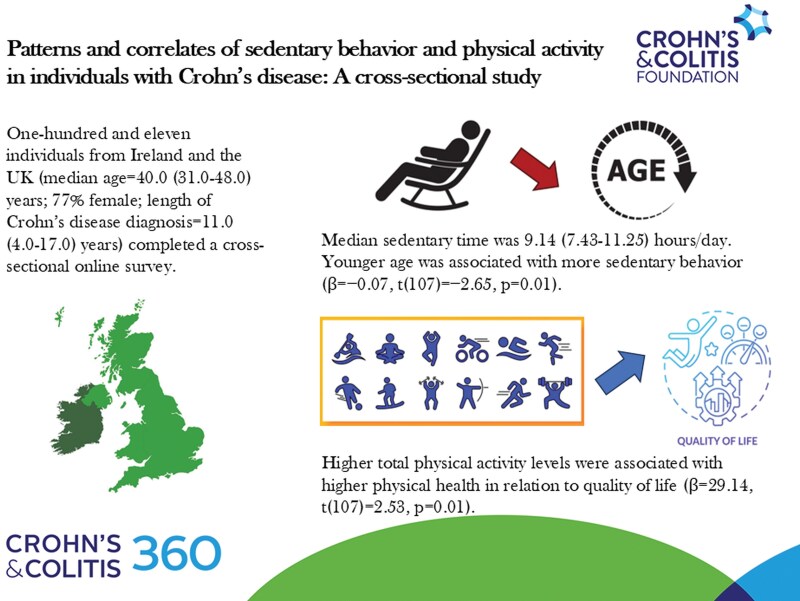

## Introduction

Crohn’s disease, a chronic inflammatory bowel disease (IBD) which can impact any section of the gastrointestinal tract, is caused by a number of factors including genetics, the environment, and altered gut microbiota, leading to unregulated immune responses.^1^ Symptomology is varied but typically includes abdominal pain, diarrhea, low-grade fever, nausea, and fatigue.^1^ Crohn’s disease has become a global disease of the 21st century, with increasing incidence in lower-middle income nations, while many higher income nations in Europe and North America have generally seen stabilization in the incidence of Crohn’s disease, although prevalence rates still exceed 0.3%.^2^ However, prevalence rates in the United Kingdom (UK) have increased between 2000 and 2017, from 220 to 400 per 100 000, according to data from The Health Improvement Network primary care database.^3^ Treatments for Crohn’s disease typically involve the use of pharmacology to control symptoms and disease progression, and when pharmacological options are not feasible, surgery is often required.^1^

Recent research has highlighted the potential therapeutic effects of lifestyle behaviors, such as increasing physical activity, with growing evidence highlighting their role in managing Crohn’s disease and its associated symptoms.^4^ For individuals with IBD, potential benefits to being more physically active include improved quality of life (QoL), cardiorespiratory fitness, bone mineral density, and immune response.^4–6^ Physical activity may also reduce anxiety and depressive symptoms, fatigue, and lower likelihood of active disease.^4–7^ However, conflicting evidence has emerged from randomized controlled trials in individuals with quiescent and mildly active IBD taking part in physical activity interventions. These trials have shown no evidence for improvements in QoL following physical activity interventions, while there has been insufficient evidence for potential benefits related to pain, fatigue, and mental health.^8^ This inconsistency is likely due to the high heterogeneity in the current research literature, demonstrating the need for further exploration in this area.

Despite the potential benefits to be gained from being more physically active, most studies show that people living with Crohn’s disease are less active than the general population, although there is some variety depending on geographical location and physical activity measurement method.^4^ For example, studies conducted in the UK,^9^ Italy,^10^ New Zealand,^11^ and Ireland^12^ have highlighted levels of inactivity ranging from 30% to 45%. Unfortunately, there are no current guidelines that provide specific physical activity recommendations to those living with Crohn’s disease. Nevertheless, the latest World Health Organization (WHO) guidelines recommend that all adults with chronic health conditions should partake in 150-300 min/week of moderate physical activity, or 75-150 min/week of vigorous physical activity, or an equivalent amount of both intensities.^13^ In addition, at least 2 days/week of strengthening activity involving all major muscle groups should be undertaken, as well as limiting the amount of sedentary behavior.^13^

To date, much of the research within the area has focused on IBD and physical activity, dually considering Crohn’s disease alongside ulcerative colitis in study cohorts. Yet, there are important clinical differences between the conditions,^14^ which means more specific investigations are warranted with regards to physical activity in Crohn’s disease. In particular, there is a need to extend this evidence base to increase certainty in how physical activity may provide benefits in terms of quality of life, mental health, and well-being to those living with Crohn’s disease.^4^ There is also a need to understand the role reducing levels of sedentary behavior might have on the health status in this specific population. This relatively new topic area has not been deeply explored with regards to Crohn’s disease, but research across various populations has highlighted that a high level of sedentary behavior is associated with poorer QoL, cognitive function, body composition, physical function, and cardiometabolic profile, respectively, alongside increased symptoms of anxiety and depression.^15^ The typical time spent in different types of sedentary behavior (eg, television viewing, office work) is currently unclear. Improving our understanding of the links between sedentary behavior and physical activity with individual and clinical characteristics will potentially inform the development of future interventions targeting increases in physical activity, reductions in sedentary behavior, or both, in those living with Crohn’s disease. Therefore, this study aimed to highlight patterns and explore the correlates of sedentary behavior and physical activity in individuals living with Crohn’s disease.

## Materials and Methods

### Study Design, Eligibility, and Recruitment

An online cross-sectional study design was utilized, using the JISC online platform. The study was advertised via Crohn’s & Colitis Ireland, Crohn’s & Colitis UK (Northern Ireland branch) and IBD Relief, Crohn’s disease support groups, the researchers’ own social media pages and word-of-mouth. Inclusion criteria included being an adult aged 18-65 years old, having a clinical diagnosis of Crohn’s disease, and residing in the United Kingdom and/or Ireland. Before completing the online survey, each participant read the participant information sheet and provided their informed consent. The survey was launched on February 6th, 2023 and was available until October 3rd, 2023. As this was an exploratory study, sample size was determined after reviewing similar research exploring physical activity in Crohn’s disease.^4^ A minimum target of ≥100 participants was set. The STROBE checklist for reporting cross-sectional studies is included in Supplementary Figure 1.

### Measurements

Demographic characteristics were collected, including age (in years); gender (female (including trans women)/male (including trans women)/nonbinary/intersex); country (Northern Ireland/Republic of Ireland/England/Scotland/Wales); ethnicity (Caucasian/Mixed ethnic background/Black African); marital status (single, never married/married or domestic partnership/widowed/divorced/separated); employment status (employed/self-employed/out of work and looking for work/out of work and not currently looking for work/homemaker/student/retired/unable to work); average household annual income (<£15 000/£15 000-£25 000/£25 000-£40 000/£40 000-£60 000/>£60 000); numbers living in the household (1/2/more than 2); smoking status (yes/no); vaping status (yes/no); currently drinking alcohol (yes/no); length of time diagnosed with Crohn’s disease (years); number of diagnosed mental health conditions; and number of diagnosed physical health conditions.

Physical activity was measured using the validated short version of the International Physical Activity Questionnaire (IPAQ-SF).^16^ Questions explored the number of days and typical time spent in vigorous intensity, moderate intensity, and walking, respectively, in the last 7 days. Metabolic equivalent minutes per week (MET min/week) were used to calculate scores for each category. Vigorous physical activity was scored as 8 METs, moderate physical activity as 4 METs, and walking as 3.3 METs.^17^ Physical activity categories were considered low, moderate, and high using previously established scoring criteria,^17^ with at least moderate scores signifying achievement of the minimum physical activity guidelines. Standard data cleaning rules were followed, whereby each activity domain (ie, vigorous, moderate, and walking) exceeding 3 hours/day were truncated to 3 hours/day.^17^ The IPAQ-SF has been utilized in other IBD populations.^9,10^

Sedentary behavior was measured using the Sedentary Behavior Questionnaire (SBQ). The SBQ asks 9 questions in relation to levels of sedentary behavior across different contexts (eg, watching television, talking on the telephone, sitting driving in a car) for a typical weekday, before asking the same questions for a typical weekend day. Responses for each question are collected on a scale which ranges from “none,” “15 min or less” and so on, up to “6 hours or more.” Typical weekday and weekend days hours/day spent sedentary were tallied separately. Daily estimates were calculated by multiplying weekday hours by 5 and weekend hours by 2, summing these together, and dividing this total number by 7 for mean hours/day spent sedentary. For these 3 summary variables, responses higher than 16 hours/day were truncated to 16 hours/day based on previous recommendations.^18^ Although the SBQ has not been previously utilized in individuals living with Crohn’s disease, it has been shown to have acceptable measurement properties.^19^

The short version of the Crohn’s Disease Activity Index (sCDAI), consisting of 3 items in relation to the number of liquid or soft stools, abdominal pain rating, and general well-being rating, was used to determine disease severity.^20^ Higher scores indicate increased Crohn’s disease severity. This questionnaire has been shown to be a valid and reliable measurement of Crohn’s disease activity^20^ and has been recently used in those with inflammatory bowel disease.^21^

The short version of the WHO Quality of Life Questionnaire (WHOQOL-BREF) was utilized to measure the quality of life across 4 domains: physical health; psychological health; social relationships; and environment, alongside single-item scores for overall QoL and general health satisfaction.^22^ The WHOQOL-BREF contains 26 items, with each question being scored on a 5-point Likert scale. Recommended scoring instructions were followed to convert the raw scores onto 0-100 points scales, with higher scores indicating higher QoL.^22^ The WHOQOL-BREF has been shown to be a cross-culturally valid assessment of QoL^23^ and has been previously utilized in individuals with IBD.^24^

The Hospital Anxiety and Depression Scale (HADS), which has been validated for use in individuals with IBD,^25^ was used to measure anxiety and depressive symptoms.^26^ It consists of 14 items, 7 in relation to anxiety symptoms and 7 in relation to depressive symptoms, with each item scored on a 0-3 point Likert scale. Higher scores reflect more symptoms of anxiety and/or depressive symptoms. A 8-10 score suggested a mild anxiety/depressive disorder, while scores ≥11 were considered probable disorder.^26^

The 7-item Short Warwick-Edinburgh Mental Well-being Scale (SWEMWBS), a revision of the 14-item WEMWBS, measured participants’ well-being.^27^ Each item is scored on a 5-point Likert scale, with better mental well-being being reflected with higher SWEMWBS scores. As recommended, raw scores were converted into metric scores.^27^ Scores ranging from 7.0 to 19.3 indicates low mental well-being, 20.0 to 27.0 indicates normal mental well-being, and 28.1 to 35.0 indicates high mental well-being, respectively.^28^ Although there appears to have been no previous studies using this particular instrument in those living with Crohn’s disease, the SWEMWBS compares well to the widely validated 14-item WEMWBS.^28^

### Data Analysis

Demographic characteristics, sedentary behavior, and physical activity intensity categories were summarized using descriptive statistics such as median (interquartile range/IQR) and number [percentage/%], unless otherwise stated. The correlates of sedentary behavior and physical activity were explored using multiple linear regression analysis, using forward selection based on likelihood ratio statistics. Dependent variables included daily time spent in sedentary behavior and MET/minutes in different intensities of physical activity (eg, IPAQ total physical activity, IPAQ vigorous physical activity, IPAQ moderate physical activity, and IPAQ walking). Independent variables included into the model were: age; gender; household income; numbers living in the household; number of mental health conditions; number of physical health conditions; sCDAI score; 4 WHOQOL-BREF domains; HADS anxiety score; HADS depression score; and the SWEMWBS metric score. Due to their single-item nature and conceptual overlap with the 4 WHOQOL-BREF domain scores, overall QoL and general health satisfaction were not included as independent variables. Due to the exploratory nature of this study, corrections for multiplicity were not made. The significance levels are therefore descriptive, rather than inferential. To explore differences between weekday and weekend day sedentary behavior, the Wilcoxon signed-rank test was utilized due to the Kolmogorov–Smirnov test determining nonparametric analyses were most appropriate. SPSS version 29.0.0 (IBM) was used for conducting statistical analyses. Statistical significance was set at *P* < .05.

### Ethical Considerations

The study was approved by the Ulster University School of Sport and Exercise Science Filter Committee on February 6th, 2023 (reference number: MG08-23).

## Results

### Demographics of the Participants

One-hundred and eleven individuals (median age = 40.0 [31.0-48.0] years; 77% female) completed the survey. In terms of missing data, 1 participant provided a distance rather than a time duration for the time spent completing moderate activities for the IPAQ. Another participant said their number of weekly stools was “400 000,” an impossible number, meaning disease severity could not be calculated for this particular individual. The majority of participants were from the island of Ireland, lived in households with 3 or more individuals and were almost exclusively Caucasian (Table 1). Seventy-one percent of participants had an average household income >£25 000. In terms of health conditions, more volunteers reported having at least 1 physical condition compared with a mental health condition. The average participant had been diagnosed with Crohn’s disease for at least a decade, with 54% having “active” Crohn’s disease status. The WHOQOL-BREF indicated that the highest QoL score from the 4 multi-item domains was for the environmental domain. Using single items from the WHOQOL-BREF to assess overall QoL and general health satisfaction, the median responses were “good” and “dissatisfied”, respectively. Almost 70% reported having at least mild symptoms of anxiety, while ~47% had at least mild depressive symptoms. Medium-to-high mental well-being was reported by 56.8% of the sample.

**Table 1.** Demographic and clinical characteristics for individuals living with Crohn’s disease (*n* = 111).

Variable	Median (IQR) or frequency [%]
Age (years)	40.0 (31.0-48.0)
**Gender**	
Male	24 [21.6]
Female	85 [76.6]
Nonbinary	2 [1.8]
**Country**	
Northern Ireland	43 [38.7]
Republic of Ireland	53 [47.7]
England	11 [9.9]
Scotland	3 [2.7]
Wales	1 [0.9]
**Ethnicity**	
Caucasian	109 [98.2]
Other background (ie, Black African or Mixed)	2 [1.8]
**Average annual household income**	
Below £15 000	15 [13.5]
£15 000-£24 999	17 [15.3]
£25 000-£39 999	27 [24.3]
£40 000-£59 999	26 [23.4]
Over £60 000	26 [23.4]
**Numbers living in household**	
One	24 [21.6]
Two	25 [22.5]
Three or more	62 [55.9]
**Number of health conditions**	
Mental conditions	0.0 (0.0-1.0)
No mental health conditions	66 [59.5]
Physical conditions (apart from Crohn’s disease)	1.0 (0.0-2.0)
No physical health conditions (apart from Crohn’s disease)	46 [41.4]
**Crohn’s disease status** ^*^	
Length of time since diagnosis (years)	11.0 (4.0-17.0)
sCDAI continuous score (0~600; higher = more activity)	166.0 (95.0-233.0)
Remission	51 [46.4]
Mild activity	28 [25.5]
Moderate-severe activity	31 [28.2]
**WHOQOL-BREF (0-100; higher = better QoL)**	
Physical health	53.6 (39.3-67.9)
Psychological health	54.2 (37.5-66.7)
Social relationships	58.3 (33.3-75.0)
Environmental	65.6 (53.1-78.1)
Overall QoL^**^	75.0 (50.0-75.0)
General health satisfaction^**^	25.0 (25.0-75.0)
**HADS (0-21; higher = more symptoms)**	
HADS anxiety symptoms continuous score	10.0 (7.0-14.0)
Non-case	34 [30.6]
Mild anxiety symptoms	23 [20.7]
Moderate anxiety symptoms	29 [26.1]
Severe anxiety symptoms	25 [22.5]
HADS depression symptoms continuous score	7.0 (3.0-10.0)
Noncase	59 [53.2]
Mild depressive symptoms	28 [25.2]
Moderate depressive symptoms	19 [17.1]
Severe depressive symptoms	5 [4.5]
**SWEMWBS (7-35; higher = better mental well-being)**	
SWEMWBS continuous score	20.0 (18.0-23.2)
Low well-being	48 [43.2]
Medium well-being	59 [53.2]
High well-being	4 [3.6]

Abbreviations: sCDAI = Short version of the Crohn’s Disease Activity Index; HADS = Hospital Anxiety and Depression Scale; SWEMWBS = Short Warwick-Edinburgh Mental Well-Being Scale; WHOQOL-BREF = Brief World Health Organization Quality of Life tool.

^*^ *n* = 1 missing for the sCDAI.

^**^Derived from a single item.

### Sedentary Behavior and Physical Activity

The average times spent in sedentary behavior and different intensities of physical activity are displayed in Table 2. For sedentary behavior, the cohort was generally more sedentary during a typical weekend day compared with a typical weekday (+2.00 median hours/day; *P* < .001). The most common physical activity was walking, with 91.9% completing some amount of walking during the week. In comparison, only 50.5% and 47.3% reported taking part in any amount of IPAQ-derived vigorous and moderate physical activities. In terms of achievement of the physical activity guidelines, just over three-quarters reported adherence.

**Table 2.** Sedentary behavior and physical activity for individuals living with Crohn’s disease (*n* = 111).

Variable	Median (IQR) or frequency [%]
SBQ weekday sedentary behavior (hours/day)	10.00 (7.50-12.00)
SBQ weekend sedentary behavior (hours/day)	8.00 (5.50-10.75)
SBQ typical daily sedentary behavior (hours/day)	9.14 (7.43-11.25)
IPAQ total physical activity (MET-min/week)	1617.00 (566.70-3093.00)
IPAQ vigorous physical activity (MET-min/week)	240.00 (0.00-1195.20)
IPAQ moderate physical activity^*^ (MET-min/week)	0.00 (0.00-565.80)
IPAQ walking (MET-min/week)	693.00 (261.36-1386.00)
**IPAQ physical activity category**	
IPAQ low category	26 [23.42]
IPAQ moderate category	55 [49.55]
IPAQ high category	30 [27.03]

Abbreviations: IPAQ = International Physical Activity Questionnaire; IQR = Interquartile range; MET-min/week = Metabolic equivalent minutes per week; SBQ = Sedentary Behavior Questionnaire.

^*^ *n* = 1 missing.

Table 3 highlights the different contexts where sedentary behavior took place on weekdays compared with weekend days. The most common sedentary behaviors completed on a typical weekday included paper/computer work, watching television, and transport-related activities. On typical weekend days, the most common sedentary behaviors included watching television and transport-related activities. Significantly more time was spent watching television (median increase = 1.00 hour/day; *P* < .001), playing computer/video games (median increase = 0.00 hours/day; *P* = .009), and reading a book/magazine (median increase = 0.00 hours/day; *P* = .001) on weekend days compared with weekdays, while the opposite was true for paper/computer work (3.75 median hours/day more during weekdays compared with weekend days; <.001).

**Table 3.** Different contexts for sedentary behavior for individuals living with Crohn’s disease (*n* = 111).

Context	SBQ weekday (median hours/day)	SBQ weekend day (median hours/day)	Differences between weekday and weekend day (*P*-value)
Watching television (including videos on VCR/DVD)	2.00 (1.00-3.00)	3.00 (2.00-4.00)	<.001^*^
Playing computer or video games	0.00 (0.00-0.00)	0.00 (0.00-0.00)	.009^*^
Sitting listening to music on the radio, tapes, or CDs	0.50 (0.00-1.00)	0.50 (0.00-2.00)	.108
Sitting and talking on the phone	0.50 (0.25-1.00)	0.50 (0.25-1.00)	.931
Doing paperwork or computer work (office work, emails, paying bills, etc.)	4.00 (0.50-6.00)	0.25 (0.00-1.00)	<.001^*^
Sitting reading a book or magazine	0.50 (0.00-1.00)	0.50 (0.00-1.00)	.001^*^
Playing a musical instrument	0.00 (0.00-0.00)	0.00 (0.00-0.00)	.655
Doing artwork or crafts	0.00 (0.00-0.00)	0.00 (0.00-0.00)	.664
Sitting and driving in a car, bus, or train	1.00 (0.50-1.00)	1.00 (0.50-2.00)	.404

Abbreviations: CD = Compact disc; DVD = Digital versatile disc; VCR = Videocassette recorder; SBQ = Sedentary Behavior Questionnaire.

^*^Statistically significant difference (*P* < .05).

### Correlates of Sedentary Behavior and Physical Activity

Table 4 highlights significant (*P* <.05) correlate variables which were included in the forward regression analysis. With only 2 participants (ie, 1.8%) identifying themselves as nonbinary, compared with larger numbers for males and females, these participants were removed for the regression analysis as the number was too small. The correlates selected in this study explained 5-10% of the variance in sedentary behavior and physical activity. For SBQ-measured sedentary behavior, the only correlate was age (β = −0.07, *t*(107) = −2.65, *P* =.01); with individuals who were more sedentary being younger. For both IPAQ total and vigorous physical activity, the WHOQOL-BREF physical health domain was the only correlate (β = 29.14, *t*(107) = 2.53, *P* =.01 and β = 23.10, *t*(107) = 3.55, *P ≤*.001, respectively), with individuals who were more active having higher self-reported physical health. For IPAQ moderate physical activity, gender was the only correlate (β = −510.54, *t*(106) = −2.68, *P* =.01); with males having higher levels of moderate physical activity. No correlates were found for IPAQ walking.

**Table 4.** Correlate variables for sedentary behavior and physical activity for individuals living with Crohn’s disease.

Dependent variable	Correlate variable	Unstandardized coefficients B(SE)	Adjusted *R*^2^	*P*-value^*^
SBQ sedentary behavior (*n* = 108)	Age	−0.07	0.05	.01
IPAQ total physical activity (*n* = 108)	WHOQOL-BREF physical health domain	29.14	0.05	.01
IPAQ vigorous physical activity (*n* = 108)	WHOQOL-BREF physical health domain	23.1	0.10	<.001
IPAQ moderate physical activity (*n* = 107)	Gender	−510.54	0.06	.01
IPAQ walking (*n* = 108)	No correlates	---	---	---

Abbreviations: IPAQ = International Physical Activity Questionnaire; *n* = number; SBQ = Sedentary Behavior Questionnaire; WHOQOL-BREF = Short version of the World Health Organization Quality of Life Questionnaire.

^*^Statistically significant (*P* < .05).

## Discussion

This study aimed to describe patterns and explore the correlates of sedentary behavior and physical activity in individuals living with Crohn’s disease, while the secondary aim was to highlight the time spent in different contexts for sedentary behavior. Almost a quarter of volunteers reported physical activity levels lower than the recommended physical activity guidelines. The most common activity was walking, with 92% in the current sample completing any amount of walking. This particular finding is unsurprising, as a number of studies in Crohn’s disease, and more generally in IBD, have found that walking was a preferred activity.^9,12^ Despite these seemingly positive results for physical activity, many participants reported excessive sedentary behavior, with many typically spending ∼9 hours/day being sedentary, with common sedentary pursuits including watching television, transport-related activity, and talking on the phone. Higher levels of total physical activity, as well as vigorous physical activity, were associated with higher QoL physical health domain scores. Being female was more associated with less moderate physical activity levels, while older age was associated with less sedentary behavior.

To help contextualize the findings linked with patterns of sedentary behavior and physical activity, we have compared our current sample with other populations with Crohn’s disease.^9–12,21,29^ Although the study samples are relatively heterogeneous in terms of the total sample size, gender profile, and cultural contexts, some interesting similarities and contrasts emerge. In terms of reported adherence to the physical activity guidelines, our cohort appears to be relatively similar to other cohorts in the USA (∼77%),^29^ New Zealand (∼70%),^11^ and the UK (73%-77% for clinical remission and mildly active disease groups).^9^ However, the current cohort appears to be more adherent to the physical activity guidelines compared with other cohorts in Italy (∼58%)^10^ and Ireland (∼55%, although this includes those with ulcerative colitis).^12^ The volume of total physical activity, in terms of MET-min/week, appears similar to a New Zealand cohort,^11^ less active compared with UK^9^ and Dutch,^21^ but more active compared with Italian^10^ and US^29^ cohorts. Most of the current studies in Crohn’s disease have used the IPAQ-SF to measure physical activity in their samples, allowing appropriate comparisons to occur. With the majority of the current cohort being from the island of Ireland, it is worth noting that only 37% of the Irish general population report meeting the physical activity guidelines,^30^ suggesting those living with Crohn’s disease are more likely to self-report meeting the physical activity guidelines. However, the general population figures were derived using a different questionnaire than the IPAQ-SF, which possibly could mean this is not a “like-for-like” comparison. Furthermore, the use of self-reported tools to measure physical activity may have resulted in an over-inflation of participants actual physical activity levels due to common limitations such as recall bias and potential misinterpretation of intensity levels.^31^ For example, a study conducted in Australian individuals living with Crohn’s disease used device-based measurement of physical activity, highlighting the median percentage of daily time spent in moderate-vigorous physical activity was only 2.3%, with typically only 1 moderate-vigorous physical activity bout lasting 10 min being measured over the week which represented 7.7 min per week across the cohort.^32^

Age was not found to be a correlate of any of the physical activity variables. This contrasts with recent research in Italian individuals with IBD, which showed older age (>50 years old) was an important risk factor for reduced levels of physical activity.^33^ Another factor not identified in the current study as a correlate of any physical activity variable was the number living in the household. Although not directly comparable with the current study, Gravina and colleagues demonstrated that partner status and social network support, particularly whether they were supportive of physical activity or not, were important factors in the activity levels in individuals with IBD.^34^ Although not demonstrated in the current study, clinicians should still consider age and social influences when encouraging individuals with Crohn’s disease to engage with physical activity.

Although mental health variables did not correlate with any of the physical activity variables in the current study, other research by Tew *et al.*^9^ has shown this to be the case for depressive symptoms but not anxiety symptoms. Higher levels of total physical activity were associated with higher QoL physical health domain scores. Although these results contrast with research conducted in New Zealand which found that physical activity was not correlated with QoL, other research conducted in a US population has found significant positive associations for physical activity with IBDQ-measured QoL.^29^ Taken together, these results demonstrate the role partaking in physical activity might have in improving QoL in individuals living with Crohn’s disease. Improving or maintaining QoL is an important global health goal across many population groups, with an umbrella review highlighting that moderate-to-strong evidence exists regarding the role physical activity has in improving QoL in adults and older adults in the general population, those with psychiatric disorders such as schizophrenia, and those with neurological conditions such as Parkinson’s disease.^35^ This is no different with IBD, with QoL being identified as an important clinical endpoint to understand how different treatment strategies may positively influence the health of individuals with such conditions.^36^ Although there is preliminary evidence of the positive role physical activity may have for those living with Crohn’s disease, in terms of improving QoL, there is a need for a larger body of research to add robustness to this evidence base.^4^ Also, it is important to consider the likely bidirectional relationship between physical activity and QoL. In a biracial cohort of middle-aged US adults, higher self-rated health at baseline was more likely to be associated with more favorable changes in total physical activity and time spent in prolonged sedentary behavior at 10-year follow-up.^37^

Few studies have attempted to measure sedentary behavior in a cohort living with Crohn’s disease. The current study estimated that during a typical weekday, ~7 median hours/day were dedicated to paper/computer work, watching television, and transport-related activities, while in total, ~>9 median hours/day were spent being sedentary during a typical day. One study conducted in an Italian cohort found lower sedentary behavior levels compared with the current study (~2.5 hours less),^10^ while another study in a UK cohort found daily time in sedentary behavior varied depending on disease severity; 6.6 hours for those in remission but up to 9.4 hours for those with severe disease activity.^9^ Although few countries have currently adopted quantitative guidelines for daily limits to sedentary behavior, including Ireland and the UK, the Canadian government have published guidelines recommending limiting sedentary time to no more than 8 hours/day, while recreational screen-time should be no more than 3 hours/day.^38^ In comparison, few in the current sample would achieve these particular guidelines. With systematic reviews highlighting the positive associations between sedentary behavior and biomarkers of ill-health (ie, insulin resistance and dyslipidemia),^39^ it is important to highlight that these are also factors linked with increased risk and progression of Crohn’s disease.^40^ Marrying these 2 aspects together, recent work by Chen and colleagues^41^ has highlighted the possible link of sedentary behavior with IBD, including Crohn’s disease. With those living with severe Crohn’s disease unlikely to be able to participate fully in moderate-vigorous physical activities,^9^ aiming to reduce sedentary behavior with any intensity of physical activity could be a new research focus in this particular topic area.

In terms of the correlates of sedentary behavior, only age was associated with sedentary behavior in the current study. In contrast to many other studies in other adult populations,^42^ the current study found that older age was associated with reduced levels of sedentary behavior. Although an unusual finding, this could potentially be explained by the job occupations of the study participants, with younger participants possibly working in more desk-based environments. With research exploring the relationships between sedentary behavior and health gathering pace, there is a need to more fully understand if there are other potential correlates of sedentary behavior in those living with Crohn’s disease, such as more objective markers of disease severity such as inflammatory biomarkers (eg, C-reactive protein) or endoscopic scoring.

Study strengths included the use of validated questionnaires for measuring physical activity, QoL, mental health, and mental well-being. This study also specifically focused on individuals living with Crohn’s disease, rather than IBD in general, and recruited a larger sample size compared with previous studies.^4^ However, information on aspects such as whether participants had a stoma bag or not would have proved useful to further understand unique aspects in relation to Crohn’s disease. It was also one of the first studies to undertake an in-depth exploration of sedentary behavior in a population living with Crohn’s disease, in terms of exploring the levels of sedentary behavior across weekday, weekend days, and different contexts in addition to exploring potential correlates.

In line with these strengths, study limitations to consider include its cross-sectional nature, meaning that cause-and-effect cannot be established. Also, generalizability to the wider UK population might be limited as the majority of the sample were from Ireland and there were also less males than females recruited, although it is worth noting that Crohn’s disease prevalence in the UK appears to be higher in females compared with males (0.44%, 95% CI: 0.43-0.45 vs 0.35%, 95% CI: 0.34-0.36, respectively).^3^ Crohn’s disease activity was also determined through subjective means, using the sCDAI, rather than objectively using blood biomarkers. Finally, dependence on self-reported sedentary behavior and physical activity measures could have been an issue.^31^

## Conclusion

To summarize, this online survey has indicated that individuals living with Crohn’s disease in Ireland and the UK may participate in high levels of physical activity and that physical activity was positively associated with higher physical QoL. This study also addressed a future research direction recommended in a recent narrative review, in terms of exploring the potential role of sedentary behavior in individuals living with Crohn’s disease.^6^ Younger age was found to be a correlate for higher levels of sedentary behavior, while individuals participated in high levels of sedentary behavior which may negatively impact on their health. With many studies in the area depending on self-report tools, there is a need for more research using device-based tools (ie, accelerometry) to measure sedentary behavior and physical activity more accurately in individuals living with Crohn’s disease, as these might be useful lifestyle variables to target for health improvement.

## Supplementary Material

otaf042_suppl_Supplementary_Figure_S1

## Data Availability

The data underlying this article will be shared on reasonable request to the corresponding author.
